# Relationship between bone union and degree of bone marrow fibrosis at resection margins of advanced mandibular ORN

**DOI:** 10.1007/s00784-024-06008-3

**Published:** 2024-11-05

**Authors:** Hiroaki Ohori, Eiji Iwata, Chihiro Ichikawa, Manabu Shigeoka, Yoshiaki Tadokoro, Daisuke Takeda, Junya Kusumoto, Takumi Hasegawa, Masaya Akashi

**Affiliations:** 1 Department of Oral and Maxillofacial Surgery, Kobe University Graduate School of Medicine, Kobe, Japan; 2 Department of Pathology, Kakogawa Central City Hospital, Kakogawa, Japan; 3 Department of Pathology, Division of Pathology, Kobe University Graduate School of Medicine, Kobe, Japan

**Keywords:** Osteoradionecrosis, Bone marrow fibrosis, Bone union, Collagen fibers, Transferred fibula flap

## Abstract

**Background:**

The pathological evaluation of cancellous bone at resection margins of mandibular osteoradionecrosis (ORN) has not been well elucidated. Here, we developed a unique classification system for evaluating the degree of bone marrow fibrosis, one of most common pathological features, in patients with mandibular ORN, based on which we investigated its relationship with treatment outcome.

**Methods:**

This study included 15 patients who underwent mandibulectomy and free fibula osteocutaneous flap reconstruction. The extent of mandibulectomy was determined, with safety margins of approximately 10 mm from the apparent osteolytic areas on preoperative computed tomography image. Special staining was performed on thin sections from center of the osteolytic areas (medial area) and bilateral resection margins, and the degree of bone marrow fibrosis was evaluated and investigated its relationship with presence of bone union as a treatment outcome.

**Results:**

The degree of bone marrow fibrosis of medial area was significantly higher than those of resection margins. Although most resection margins had collagen fibers which indicate severe fibrosis, all transferred fibula flaps achieved bone union.

**Conclusion:**

When mandibulectomy is performed with safety margins of approximately 10 mm from the apparent osteolytic areas, all transferred fibula flaps achieved bone union regardless of the degree of bone marrow fibrosis at resection margin. In other words, the association between severe bone marrow fibrosis at resection margins and treatment outcome was not seen.

**Clinical Relevance:**

Setting safety margins of approximately 10 mm may achieve bone union, but further study is needed.

## Introduction

Osteoradionecrosis (ORN) of the jaw is a severe and refractory complication, following radiation therapy (RT) for head and neck cancer that develops in approximately 2–22% of the case [1]. The patients with irradiated mandibles exposed to cumulative have greater than 60 Gy appear to be at highest risk [2]. Early or limited ORN of the jaw can be treated with conservative therapies including improvements in oral health, curettage, debridement, long-term antibiotic use, and hyperbaric oxygen therapy. However, advanced mandibular ORN with radiographically evident osteolysis of the inferior border, pathological fracture, or oro-cutaneous fistula is unlikely to respond to conservative therapy [3]. These advanced cases are best treated by mandibular resection, including hemi- and segmental mandibulectomy and reconstruction with a vascularized free flap, particularly free fibular bone flaps [3–5]. Although setting the extent of resection and pathological evaluation of resection margins are essential in mandibular resections, no treatment guidelines or position papers for ORN of the jaw have been established. Therefore, the criteria for pathological evaluation of resection margins are unclear. Several studies have focused on the presence of residual necrotic bone at resection margins [6–8]. Zaghi et al. reported that the presence of residual necrotic bone at the resection margins of segmental mandibulectomies did not correlate with ORN recurrence [6]. We reported that complete extirpation of necrotic bone (especially cortical bone) is sometimes impossible, even if resection of the destroyed bone area has a wide safety margin [7]. However, the pathological evaluation of cancellous bone (i.e., bone marrow) at resection margins has not been well elucidated.

Radiation-induced fibrosis (RIF) is an unavoidable pathological process that occurs in the skin, subcutaneous tissue, lungs, and gastrointestinal tract, as well as in almost every part of the body that is exposed to irradiation and lasts for a long time after RT [9]. Skin induration, muscle shortening, lymphedema, ulceration, and pain are known [10]. Locally specific symptoms include trismus, xerostomia, dysphagia, and ORN in patients with head and neck cancer. RIF is involved in the pathological development, maintenance, and progression of ORN and the main event in ORN progression is the activation and dysregulation of fibroblastic activity [10]. Several histological studies have reported bone marrow fibrosis as one of the most common pathological features of human mandibular ORN [11–13]. The degree of bone marrow fibrosis in each part of the jaw bone may represent the ORN severity. We hypothesized that severe bone marrow fibrosis at resection margins, which indicates remaining of severe ORN in mandible, have association with treatment outcome. In the present study, we developed a unique classification system for evaluating the degree of bone marrow fibrosis in patients with mandibular ORN, based on which we investigated its relationship with bone union after free fibula osteocutaneous flap reconstruction.

## Patients & methods

### Patients

**Fig. 1:**
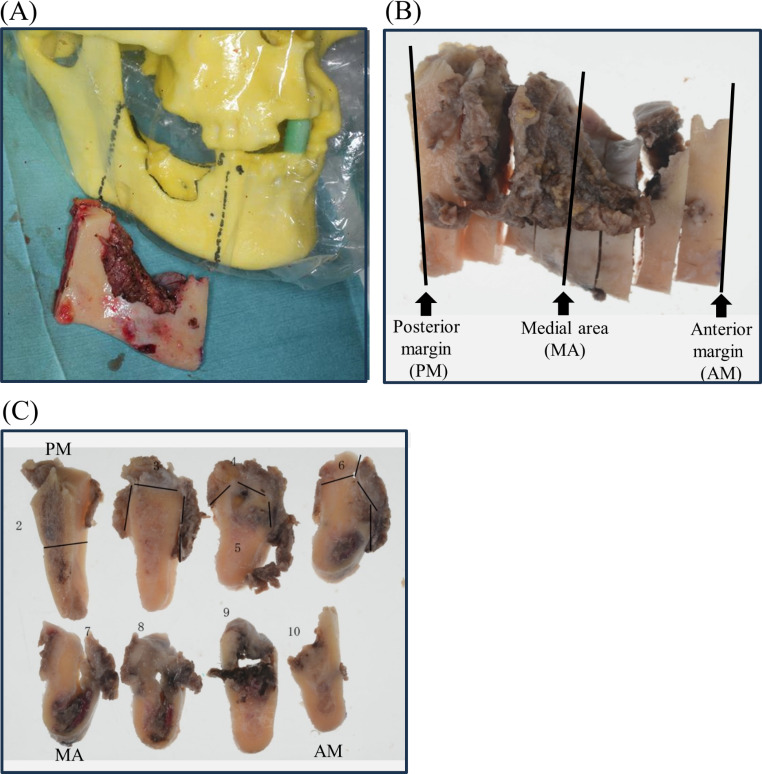
A resected bone specimen.(**A**) The 3D-printed CT model and resected bone specimen. (**B**) (**C**) Three parts of the bone specimen, including the anteroposterior margins (AM and PM) and medial area (MA), were subjected to histological analysis. AM represents anterior margin; MA represents medial area; PM represents posterior margin

The present study included 15 patients who were diagnosed with mandibular ORN and underwent hemi- or segmental mandibulectomy and free fibula osteocutaneous flap reconstruction at the Department of Oral and Maxillofacial Surgery, Kobe University Hospital between January 2014 and December 2019. The most accepted definition of ORN is irradiated bone that becomes devitalized and is exposed through the overlying skin or mucosa, without tumor recurrence, and does not heal within 3 months and we also followed this [14]. In addition to pathological fractures, severe pain caused by damage to the inferior alveolar nerve is regarded as an important finding that guides the decision to intervene surgically. The extent of mandibulectomy, with safety margins of approximately 10 mm from the apparent osteolytic areas, was determined by using preoperative computed tomography (CT) (Fig. 1A). Patients whose follow-up duration was less than 2 years after surgery, those who presented with thrombosis at the anastomosis site, those who had early tumor recurrence, and those with missing data that were needed in this study were excluded.

### Data collection

The following epidemiological data were collected retrospectively from the patients’ medical records: sex; age; medical history including diabetes mellitus (DM) and chronic kidney disease (CKD); tumor site; reconstruction after tumor resection; type of RT (conventional or intensity modulated radiation therapy [IMRT]); total radiation dose; interval between RT completion date and surgery; extent of mandibulectomy (segmental or hemi); and presence of bone union between fibula flap and residual mandible. In addition, the positional relationship between the resection margins and radiation range was investigated in patients whose CT image data with the radiation dose distribution map were obtained. “High dose of irradiation” was defined as more than 50 Gy and “low dose of irradiation” as less than 50 Gy [15]. Presence of bone union between fibula flap and residual mandible was judged using panoramic radiograph image and CT image ≥ 2 years after surgery, as previously reported [16]. 

### Classification of bone marrow fibrosis

**Fig. 2:**
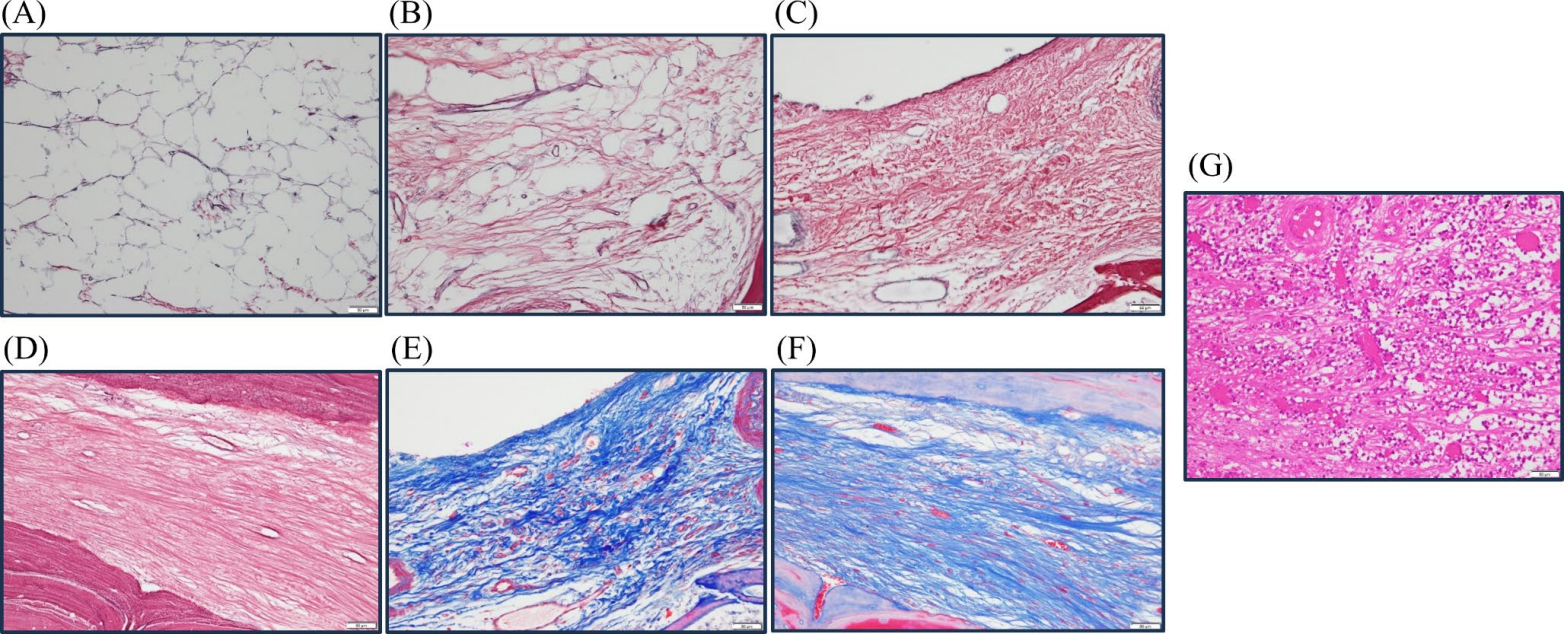
Classification of bone marrow fibrosis. Silver impregnation staining of (**A**) Grade 0, (**B**) Grade 1, (**C**) Grade 2, and (**D**) Grade 3. MT staining of (**E**) Grade 2 and (**F**) Grade 3. (**G**) HE staining of inflammation

The 2016 World Health Organization (WHO) classification of myelofibrosis is a well-known grading system for bone marrow fibrosis [17]. The classification consists of four myelofibrosis grades; Grade 0: scattered linear reticulin with no intersections (cross overs) corresponding to normal bone marrow; Grade 1, loose network of reticulin with many intersections, especially in perivascular areas; Grade 2, diffuse and dense increase in reticulin with extensive intersections, occasionally with focal bundles of thick fibers mostly consistent with collagen, and/or focal osteosclerosis; Grade 3, diffuse and dense increase in reticulin with extensive intersections and coarse bundles of thick fibers consistent with collagen, usually associated with osteosclerosis (Table 1). These were evaluated using silver impregnation staining. For Grade 2 and 3, an additional Masson’s trichrome (MT) staining is recommended [17]. This classification is often used to evaluate primary myelofibrosis (PMF), which is a hematopoietic tumor. As hematopoietic tumor and ORN of the jaw are different pathologies, we newly added a new grading “inflammation” as a unique classification of ORN of the jaw in the present study (Table 2). This grading was defined as granulation tissue with infiltration of inflammatory cells. In general, hematoxylin-eosin (HE) staining is recommended to value inflammatory cells. The histopathological images per grade are depicted in Fig. 2.

**Table 1:** The 2016 WHO classification of myelofibrosis

Myelofibrosis grading	
Grade 0	Scattered linear reticulin with no intersections (crossovers) corresponding to normal bone marrow
Grade 1	Loose network of reticulin with many intersections, especially in perivascular areas
Grade 2	Diffuse and dense increase in reticulin with extensive intersections, occasionally with focal bundles of thick fibers mostly consistent with collagen, and/or focal osteosclerosis*
Grade 3	Diffuse and dense increase in reticulin with extensive intersections and coarse bundles of thick fibers consistent with collagen, usually associated with osteosclerosis*

*In Grade 2 or Grade 3, an additional trichrome stain is recommended

**Table 2:** Our unique classification of bone marrow fibrosis

Bone marrow fibrosis grading	
Grade 0	Scattered linear reticulin with no intersections (crossovers) corresponding to normal bone marrow
Grade 1	Loose network of reticulin with many intersections, especially in perivascular areas
Grade 2	Diffuse and dense increase in reticulin with extensive intersections, occasionally with focal bundles of thick fibers mostly consistent with collagen, and/or focal osteosclerosis*
Grade 3	Diffuse and dense increase in reticulin with extensive intersections and coarse bundles of thick fibers consistent with collagen, usually associated with osteosclerosis*
Inflammation (new grading)	Granulation tissue with infiltration of inflammatory cells **

*In Grade 2 or Grade 3, an additional trichrome stain is recommended

**In inflammation, HE staining is recommended

### Histological analysis

Each specimen was decalcified and fixed in formalin but not frozen. The details of the decalcification method are as follows. Formic acid (98%) (FUJIFILM Wako Pure Chemical Corporation, Osaka, Japan) was diluted to 10% with distilled water. Bone specimens were immersed in 10% formic acid with ion-exchange resin and treated by ultrasonic histoprocessor (Histra-DC; Jokoh, Tokyo, Japan) for several weeks. The bone specimens were cut in approximately 1.5 cm blocks and embedded in paraffin. To evaluate the degree of bone marrow fibrosis, thin sections were obtained and stained with HE, MT (Tokyo Chemical Industry Co., Ltd, Tokyo, Japan), and silver impregnation (FUJIFILM Wako Pure Chemical Corporation, Osaka, Japan) according to the manufacturer’s protocols and imaged under bright-field illumination using a BX53 microscope (Olympus, Tokyo, Japan). Collagen appeared pink and blue under silver impregnation and MT staining, respectively.

Three parts of the bone specimen, including the anteroposterior margins and medial area, were subjected to histological analysis (Fig. 1B, C), as previously reported [7, 8]. In patients received hemi-mandibulectomy, posterior resection margin was excluded. The “medial area” was defined as center of the osteolytic areas [Fig. 1B, C] [7, 8]. Based on our classification, the grading of bone marrow fibrosis was evaluated and proportions of the surface area of each grade in the surface area of cancellous bone was investigated using the ImageJ software, version 1.47 (National Institutes of Health, Bethesda, MD, USA)　(http://rsbweb.nih.gov/ij/download.html).

### Statistical analysis

All statistical analyses were conducted using Ekuseru-Toukei 2016 (Social Survey Research Information Co., Ltd.; Tokyo, Japan). Comparison of the degree of bone marrow fibrosis in each part was examined using Fisher’s exact test. Statistical significance was established at *P* < 0.05.

## Results

Table 3 shows patients’ characteristics and treatment outcome. The median age and radiation dose were 69.0 years (range, 56–80 years) and 66.0 Gy (range, 60–81 Gy), respectively. Five patients (33.3%) were compromised hosts with medical history including DM and CKD. Over half the tumor sites were in the oropharynx, and 5 patients (33.3%) underwent reconstruction after tumor resection. The most common type of RT which patients underwent was conventional, and not IMRT. While 4 patients (26.6%) underwent hemi-mandibulectomy and free fibula osteo-cutaneous flap reconstruction, others (73.4%) received segmental mandibulectomy and the same reconstruction. As a treatment outcome, all transferred fibula flaps achieved bone union. The median follow-up period after surgery was 72 months (range, 25–112 months) (data not shown).

**Table 3:** Patients’ characteristics and treatment outcome

Case No.	Sex	Age	Medical history	Tumor site	Reconstruction after tumor resection (flap type)	Type of RT/ total dose (Gy)	Interval between RT and surgery (months)	Extent of mandibulectomy	Bone union
1	Male	57	DM	Oropharynx	No	Conventional/66	108.5 ± 5.5^a^	Segmental	+
2	Female	80	DM	Mandibular gingival	Yes (FAF)	IMRT/60	6	Segmental	+
3	Male	65		Hypopharynx	No	Conventional/60	121	Segmental	+
4	Male	63		Nasopharynx	No	Conventional/70	56	Segmental	+
5	Male	74		Oropharynx	Yes (RAM)	Conventional/66	71	Segmental	+
6	Male	78	DM	Oropharynx	Yes (Tongue flap)	Conventional/60	26	Segmental	+
7	Male	56		Oropharynx	No	Conventional/70	75	Hemi	+
8	Male	69		Oropharynx	No	Conventional/66	236.5 ± 5.5^a^	Segmental	+
9	Male	76		Nasopharynx	No	Conventional/70	201	Hemi	+
10	Male	70	CKD	Neck (unknown primary)	No	Conventional/66	75	Segmental	+
11	Male	65		Oropharynx	Yes (FAF)	Conventional/60	137	Segmental	+
12	Male	64		Oropharynx	No	Conventional/81	152	Segmental	+
13	Male	56		Oropharynx	No	Conventional/70	44	Segmental	+
14	Male	70	DM	Oropharynx	No	IMRT/69.96	43	Hemi	+
15	Male	74		Tongue	Yes (FAF)	IMRT/69.96	37	Segmental	+

^a^For 2 patients, data on the date of RT completion were not available but the year was available

DM: diabetes mellitus; CKD: chronic kidney disease; FAF: free forearm flap; and RAM: free rectus abdominis myocutaneous flap

**Fig. 3:**
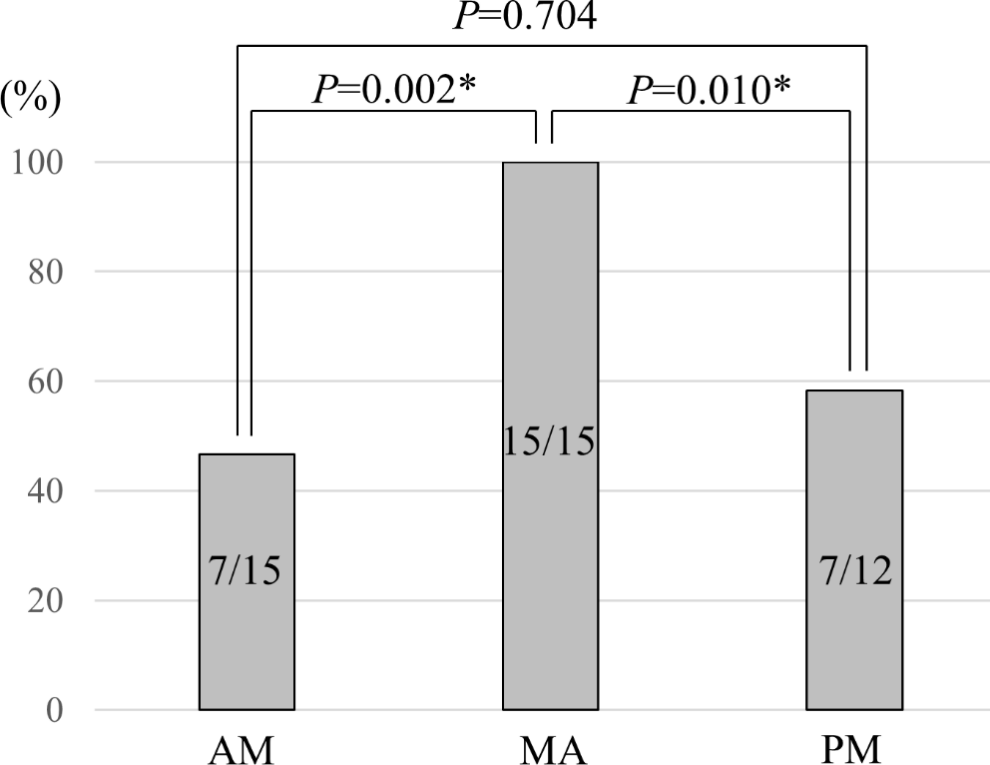
Comparison of the rate of cases with Grade 3 in each. *P < 0.05. AM represents anterior margin; MA represents medial area; PM represents posterior margin.

Table 4 shows histopathological results of the bone specimens. In medial area, all patients had collagen fibers (mainly Grade 3) and almost all (14 out of 15; 93.3%) had no normal bone marrow (Grade 0). In a total of 27 resection margins, 23 margins (85.2%) had collagen fibers (mainly Grade 2) and 12 margins (44.4%) had normal bone marrow. Interestingly, all parts which had grading “inflammation” had collagen fibers (mainly Grade 3). Figure 3 shows the comparison of the rate of cases with Grade 3 in each part. The rate of cases with Grade 3 of medial area (100.0%) was significantly higher than those of resection margins. Between the rate of cases with Grade 3 in anteroposterior margins, no significant difference was seen (anterior margin vs. posterior margin; 46.7% vs. 58.3%).

**Table 4:** Histopathological results of three parts of the bone specimens

Case No.	Part	Grade 0	Grade 1	Grade 2	Grade 3	Inflammation	Collagen fiber (≥ Grade 2)	Cases with Grade 3
1	Anterior margin Medial area Posterior margin	0% 0% 0%	0% 0% 0%	10.2% 0% 21.1%	69.7% 29.8% 78.9%	20.1% 70.2% 0%	+ + +	+ + +
2	Anterior margin Medial area Posterior margin	0% 0% 9.8%	38.8% 0% 80.1%	61.2% 0% 10.1%	0% 22.1% 0%	0% 77.9% 0%	+ + +	- + -
3	Anterior margin Medial area Posterior margin	9.7% 0% 0%	60.1% 0% 10.2%	30.2% 0% 20.1%	0% 20% 69.7%	0% 80% 0%	+ + +	- + +
4	Anterior margin Medial area Posterior margin	0% 0% 0%	0% 0% 0%	50.1% 0% 100%	49.9% 20.2% 0%	0% 79.8% 0%	+ + +	+ + -
5	Anterior margin Medial area Posterior margin	0% 0% 0%	50.2% 9.7% 100%	44.7% 20.4% 0%	0% 30.1% 0%	5.1% 39.8% 0%	+ + -	- + -
6	Anterior margin Medial area Posterior margin	60.5% 0% 68.1%	29.4% 0% 31.9%	0% 8.8% 0%	0% 62.1% 0%	10.1% 29.1% 0%	- + -	- + -
7	Anterior margin Medial area Posterior margin	0% 0% NA	0% 0% NA	0% 0% NA	30.6% 51.6% NA	69.4% 48.4% NA	+ + NA	+ + NA
8	Anterior margin* Medial area Posterior margin	20.2% 0% 100%	19.7% 19.4% 0%	10.2% 30.4% 0%	24.3% 10.0% 0%	25.6% 40.2% 0%	+ + -	+ + -
9	Anterior margin Medial area Posterior margin	0% 0% NA	79.8% 0% NA	20.2% 0% NA	0% 9.7% NA	0% 90.3% NA	+ + NA	- + NA
10	Anterior margin Medial area Posterior margin	0% 0% 0%	50.2% 0% 0%	49.8% 0% 20.2%	0% 59.5% 39.5%	0% 40.5% 40.3%	+ + +	- + +
11	Anterior margin Medial area Posterior margin	30.2% 4.7% 10.2%	37.7% 5.2% 9.7%	32.1% 90.1% 75.1%	0% 0% 0%	0% 0% 5.0%	+ + +	- - -
12	Anterior margin Medial area Posterior margin	0% 0% 0%	4.7% 0% 0%	50.2% 0% 30.3%	45.1% 29.7% 49.6%	0% 70.3% 20.1%	+ + +	+ + +
13	Anterior margin Medial area Posterior margin	9.8% 0% 0%	30.1% 0% 48.7%	60.1% 10.2% 50.3%	0% 49.6% 0%	0% 40.2% 0%	+ + +	- + -
14	Anterior margin Medial area Posterior margin	9.2% 0% NA	79.4% 0% NA	11.4% 0% NA	0% 35.9% NA	0% 64.1% NA	+ + NA	- + NA
15	Anterior margin Medial area Posterior margin	12.0% 0% 41.4%	33.0% 0% 13.5%	55.0% 7.5% 45.1%	0% 24.5% 0%	0% 68.0% 0%	+ + +	- + -

Presence of collagen fiber which indicates severe fibrosis means presence of Grade 2 or Grade 3

NA: not available

*At the anterior resection margin of one patient (No.8), partial resection was added after mandibulectomy. Thus, histopathological result may not be perfectly matched

Table 5 shows the relationship between the histopathological results of both resection margins and radiation range in 5 patients who could obtain CT image data with the radiation dose distribution map from their medical records. Three out of the 5 patients (No. 2, 7, and 15) experienced high dose of irradiation (≥ 50 Gy) to both resection margins and 5 all resection margins showed collagen fibers. In 2 out of the 5 patients (Nos. 6 and 14), the anterior margin received a low dose of irradiation (< 50 Gy); however, the posterior margin was outside the radiation range. Interestingly, although one patient had collagen fiber at resection margin (48 Gy), the other patient had no collagen fibers in either resection margin (0 Gy and 40 Gy).

**Table 5:** The relationship between histopathological results of both resection margins and radiation range

Case No.	Part	Grade 0	Grade 1	Grade 2	Grade 3	Collagen fiber (≥ Grade 2)	Extent of mandibulectomy	Relationship with radiation range	Radiation dose of the part (Gy)
2	Anterior margin Posterior margin	- +	+ +	+ +	- -	+ +	Segmental	Within Within	57 57
6	Anterior margin Posterior margin	+ +	+ +	- -	- -	- -	Segmental	Within Outside	40 0
7	Anterior margin Posterior margin	- NA	- NA	- NA	+ NA	+ NA	Hemi	Within Within	50 60
14	Anterior margin Posterior margin	+ NA	+ NA	+ NA	- NA	+ NA	Hemi	Within Outside	48 0
15	Anterior margin Posterior margin	+ +	+ +	+ +	- -	+ +	Segmental	Within Within	69.96 69.96

Presence of collagen fiber which indicates severe fibrosis means presence of Grade 2 or Grade 3

**Fig. 4:**
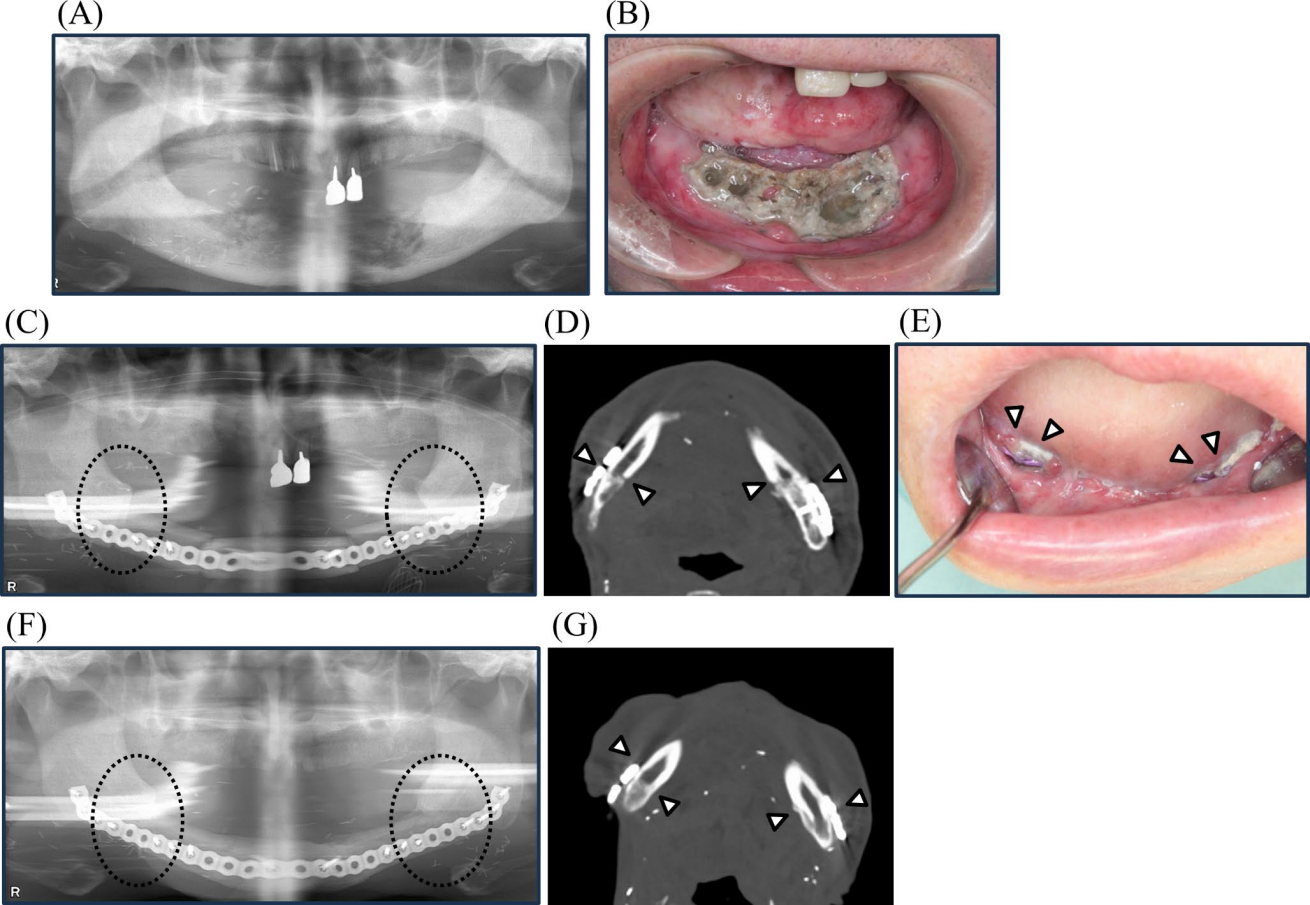
Radiological images and oral photo of a patient (No.15). (**A**)Panoramic radiograph image with advanced osteolysis before surgery. (**B**) Intraoral photo with bone exposure before surgery. The arrows show the part of bone exposure. (**C**) (**D**) Panoramic radiograph image at 2 weeks after surgery and CT image at 10 months after surgery. The circles and arrows show the bone joints of fibula flap and residual mandibles, which do not achieve bone union yet. (**E**) Intraoral photos with plate exposure at 8 days after surgery. The arrows show the part of plate exposure. (**F**) (**G**) Panoramic radiograph image and CT image at 4 years after surgery. The circles and arrows show the part of bone union

**Fig. 5:**
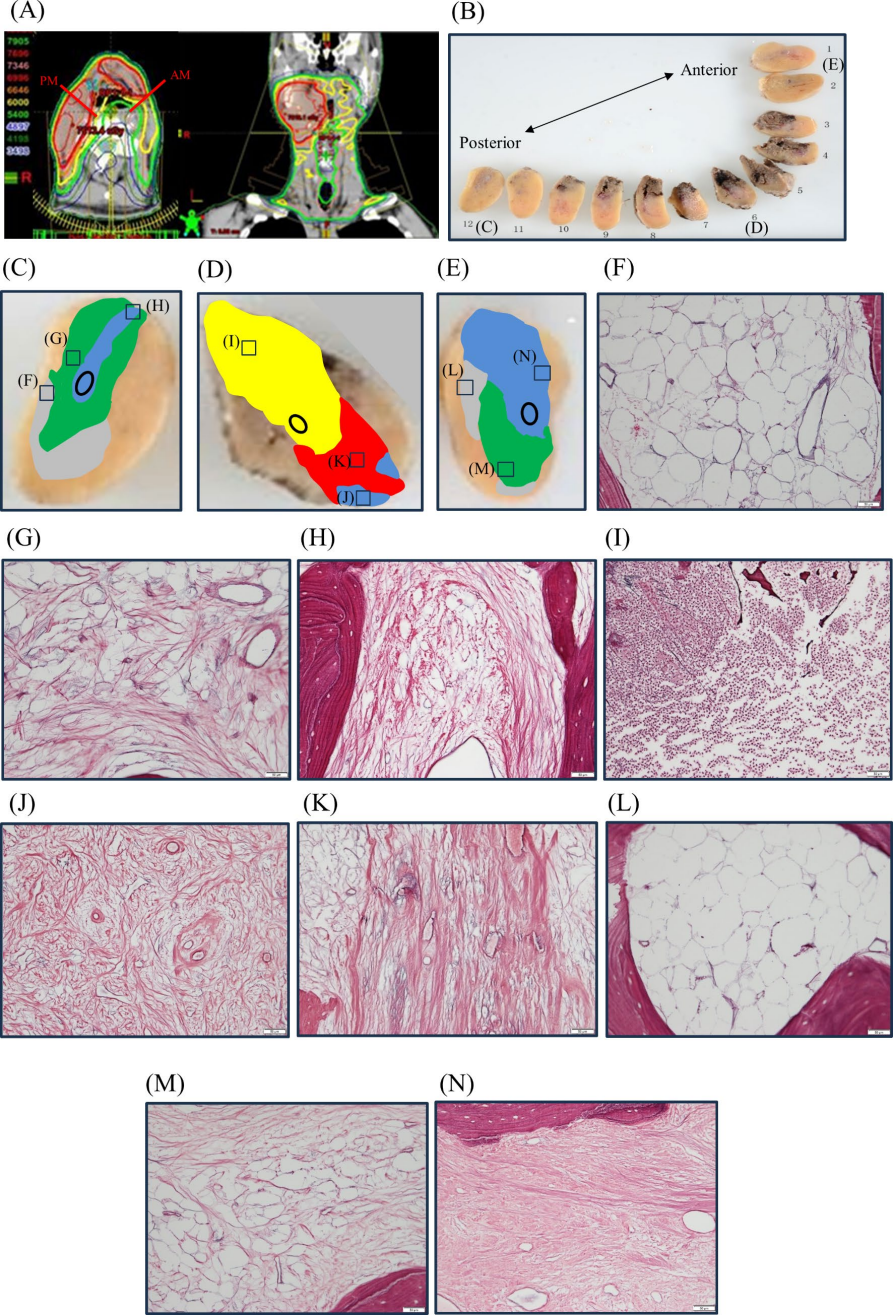
Histopathological image of a male patient (No. 15).(**A**) Axial and coronal CT image with radiation dose distribution map and both resection margins. Both resection margins were within the radiation range. (**B**) Macroscopic image of segmental mandibulectomy. (**C**) The cancellous bone of posterior margin was Grade 0 (part of gray), Grade 1 (part of green), and Grade 2 (part of blue). The circle indicates mandibular canal. (**D**) The cancellous bone of medial area was Grade 2 (part of blue), 3 (part of red), and inflammation (part of yellow). The circle indicates mandibular canal. (**E**) The cancellous bone of anterior margin was Grade 0 (part of gray), 1 (part of green), and 2 (part of blue). The circle indicates mandibular canal. Silver impregnation staining of (**F**) Grade 0, (**G**) Grade 1, and (**H**) Grade 2 in posterior margin, (**I**) Grade 2, (**J**) Grade 3, and (**K**) Inflammation in medial area, (**L**) Grade 0, (**M**) Grade 1, and (**N**) Grade 2 in anterior margin

We introduce one patient in detail. A 74-year-old male (No. 15) with right tongue cancer underwent hemi-glossectomy, free radial forearm flap reconstruction, and ipsilateral supra-omohyoid neck dissection. As postoperative adjuvant therapy, he received RT (69.96 Gy) combined with cisplatin chemotherapy. One year after RT completion, an anterior mandibular ORN developed. Although the patient received conservative therapy, including antibiotic administration and curettage, the ORN progressed (Fig. 4A, B). He complained of severe pain and was unable to sleep. The patient underwent a segmental mandibulectomy and free fibula osteocutaneous flap reconstruction 37 months after RT (Fig. 4C, D). Within a month of surgery, bone and plate exposure occurred because of wound dehiscence (Fig. 4E). Infection became protracted and he received debridement and antibiotics several times. Although the infection continued to occur repeatedly, it gradually subsided without plate removal after 3 years. In this case, both resection margins were within the radiation range and received irradiation at 69.96 Gy same as medial area (Fig. 5A). The degree of bone marrow fibrosis of medial area was Grade 2–3 and grading “inflammation”, which showed a mixture of severe fibrosis and granulation tissue with infiltration of inflammatory cells. The degrees of bone marrow fibrosis of both resection margins were Grade 0–2, which showed a mixture of normal bone marrow and severe fibrosis (Fig. 5B-N). Bone union between the fibula flap and the margins of the residual mandible was confirmed 4 years after surgery on panoramic radiograph and CT (Fig. 4F, G).

## Discussion

Mandibular resection and reconstruction with free fibula osteocutaneous flap are the best treatments for advanced mandibular ORN [3, 18]. However the pathological evaluation of cancellous bone at resection margins has not been well elucidated. We hypothesized that severe bone marrow fibrosis at resection margins, which indicates remaining of severe ORN in mandible, have association with treatment outcome. In this study, the extent of mandibulectomy was determined, with safety margins of approximately 10 mm from the apparent osteolytic areas on preoperative computed tomography image. Hence, we developed a unique classification system for evaluating the degree of bone marrow fibrosis in patients with mandibular ORN, based on which we investigated its relationship with presence of bone union. As a result, all transferred fibula flaps achieved bone union regardless of the degree of bone marrow fibrosis at resection margin.

Mandibular reconstruction has evolved tremendously with the advent of microvascular techniques and vascularized bone grafts are widely recognized as the most reliable method for mandibular reconstruction [19]. That also applies to irradiated mandible and particularly free fibula osteocutaneous flap is the most commonly used method of mandibular reconstruction for the treatment of advanced ORN [4, 5]. Disa et al. reported that 93% of the transferred fibula flaps for mandibular reconstruction, including irradiated mandible, achieved bone union [20]. In the present study, all transferred fibula flaps achieved bone union regardless of the degree of bone marrow fibrosis at resection margin. This means that even if the resection margins have severe fibrosis surgeons may not have to perform additional resection because the transferred fibula flap has a high probability of achieving bone union. However, this approach may be limited to cases involving reconstruction. The effects of the degree of bone marrow fibrosis of the resection margins in cases with marginal-mandibulectomy or with segmental mandibulectomy without reconstruction, on treatment outcomes are future tasks. In addition, determining the extent of resection of advanced mandibular ORN remains an unresolved problem. We have unified the treatment policy in our facility that the extent of mandibulectomy with safety margins of approximately 10 mm from the apparent osteolytic areas on the preoperative CT images. It may be insufficient that 10 mm of surgical margin for resection should be considered a standard from our results because of small population. Our study was nothing more than to have investigated the relationship between bone union and the degree of bone marrow fibrosis at resection margins when mandibulectomy with safety margins of approximately 10 mm from the apparent osteolytic areas on the preoperative CT images. In addition, we found that no association between severe bone marrow fibrosis at resection margins and bone union. Our results suggest that further small safety margin may be better because the flap volume could also be reduced. Further study is needed.

Although several theories on the pathophysiology of ORN have been proposed, the fibro-atrophic theory was advocated by Delanin and Lefaix [9]. It consists of three clinical and histopathological phases (a pre-fibrotic-specific inflammatory phase, a constitutive fibrotic cellular phase, and a matrix densification and remodeling phase, possibly ending in terminal tissue necrosis). From a pathological evaluation of an irradiated human bone specimen, Marx et al. found an absence of fat cells in the bone marrow of the ORN of the jaw and that were replaced with collagen [11]. The 2016 WHO classification of myelofibrosis is well-known for a grading of bone marrow fibrosis [17]. In the present study, we developed a unique classification to evaluate the degree of bone marrow fibrosis of mandibular ORN with reference to this classification. In medial area, all patients had collagen fibers (mainly Grade 3) and almost all had no normal bone marrow (Grade 0). In other hand, most margins (85.2%) had collagen fibers (mainly Grade 2) and about half (44.4%) had normal bone marrow. As a result, the degree of bone marrow fibrosis of medial area was significantly higher than those of resection margins. These suggest that the degree of bone marrow fibrosis in each part of the jaw bone represents the ORN severity in the part. Therefore, our classification may be useful for pathological evaluation of the degree of bone marrow fibrosis in ORN of the jaw. This classification may also be useful for evaluating not only mandibular ORN, but also bone marrow fibrosis of osteomyelitis of the jaw or medication-related osteonecrosis of the jaw. However, inflammation that we added as a new grading is a different pathological condition from bone marrow fibrosis in ORN. We had to distinguish the part of “real” bone marrow from the part of granulation tissue which were replaced from bone marrow to evaluate the degree of bone marrow fibrosis in cancellous bone of mandibular ORN. The role of inflammation in ORN progression is future task.

Finally, we considered the factors that cause severe bone marrow fibrosis in ORN of the jaw. RIF usually occurs 4–12 months after RT and progresses over several years [21]. In a qualitative assessment of biopsies taken from patients irradiated for oral malignancies, Marx reported progressive fibrosis over time after irradiation [22]. In the present study, median interval between RT completion and surgery was 75.0 months (range, 6–236.5 ± 5.5 months). The duration between surgery and decalcification completion of resected bone specimen also should be considered. Thus, the time elapsed after RT may be a factor that causes severe bone marrow fibrosis. Zhuang et al. reported a dose-effect relationship between the impact of RIF and the progression of ORN of the jaw; in other words, radiation dose increases the impact of RIF and may be stronger [10]. Thus, we investigated the positional relationship between the resection margins and radiation range. Three out of the 5 patients experienced high dose of irradiation (≥ 50 Gy) to both resection margins, and 5 all resection margins showed collagen fiber which indicates severe fibrosis. In other 2 out of 5 patients, the anterior margin was received a low dose of irradiation (< 50 Gy); however, the posterior margin was outside the radiation range. Interestingly, although one patient had collagen fiber at resection margin (48 Gy), the other patient had no collagen fibers in either resection margin (0 Gy and 40 Gy). These results suggest that a high dose of irradiation causes excessive bone marrow fibrosis; however, there might also be other factors. Curi et al. investigated 40 bone specimens from human mandibular ORN and found a significant increase in fibrosis in irradiated bone specimens compared to that in bone specimens [12]. Dekker et al. investigated 15 bone specimens from irradiated human mandibles and found no significant differences in bone marrow fibrosis between irradiated bone specimen and non-irradiated bone specimens [13]. A recent review showed that bone marrow fibrosis develops following infection and tissue damage, including radiation [23]. We have previously reported the possibility of bacterial spread to the mandibular condyle in human bone specimens from patients with mandibular ORN who underwent hemi-mandibulectomy [24]. We also reported that mandibular pathological fracture (i.e., progression of ORN of the jaw) could be induced by the accumulation of infection in the irradiated bone [25]. In the present study, all bone specimens with grading “inflammation”, which indicates granulation tissue with infiltration of inflammatory cells, had collagen fiber (particularly Grade 3). Thus, severe bone marrow fibrosis in the ORN of the jaw can be induced by the accumulation of infection over time in the irradiated jawbone.

This study had some limitations. First, although conventional RT is the most common type of RT, IMRT is currently the mainstream treatment. Second, we could not obtain CT images with radiation dose distribution maps from medical records of many patients because they had been treated long ago or at other hospitals. However, the main purpose of the present study was to develop a pathological classification to evaluate the degree of bone marrow fibrosis of the ORN of the jaw and to investigate the relationship between the degree of bone marrow fibrosis of the resection margins based on this classification and bone union. Finally, the population evaluated in this study were small and might have introduced biases in data selection and analyses. In future, prospective study with larger sample sizes and more detailed examinations including the roles of type of collagen fibers and inflammation should be conducted.

In conclusion, to the best of our knowledge, this is the first study to investigate the relationship between bone union and the degree of bone marrow fibrosis at resection margins of the patients with advanced mandibular ORN. When mandibulectomy is performed with safety margins of approximately 10 mm from the apparent osteolytic areas, all transferred fibula flaps achieved bone union regardless of the degree of bone marrow fibrosis at resection margin. In other words, the association between severe bone marrow fibrosis at resection margins and treatment outcome was not seen.

## Data Availability

No datasets were generated or analysed during the current study.
